# In vivo evaluation of microglia activation by intracranial iron overload in central pain after spinal cord injury

**DOI:** 10.1186/s13018-017-0578-z

**Published:** 2017-05-18

**Authors:** Fan Xing Meng, Jing Ming Hou, Tian Sheng Sun

**Affiliations:** 1 Third Military Medical University, No. 30 Gaotanyan Street, Chongqing, 400038 China; 2 Department of Orthopedics, Chinese PLA Army General Hospital, Dongcheng District, Nanmencang No. 5, Beijing, 100700 China; 3 Department of Rehabilitation, Southwest Hospital, Third Military Medical University, No. 30 Gaotanyan Street, Chongqing, 400038 China

**Keywords:** Central pain, Spinal cord injury, Iron, Microglia, NF-κB

## Abstract

**Background:**

Central pain (CP) is a common clinical problem in patients with spinal cord injury (SCI). Recent studies found the pathogenesis of CP was related to the remodeling of the brain. We investigate the roles of iron overload and subsequent microglia activate in the remodeling of the brain after SCI.

**Methods:**

An SCI-induced CP model was established in Sprague-Dawley rats that were randomly assigned to SCI, sham operation, deferoxamine (DFX), minocycline, and nitric oxide synthase inhibitor treatment groups. At 12 weeks, pain behavior and thermal pain threshold were evaluated in each group, and *iron transferrin receptor* (*TfR*)*1* and *ferritin* (*Fn*) mRNA, as well as iron-regulatory protein (IRP)1, FN, lactoferrin, and nuclear factor (NF)-κB protein levels in the rat brains were measured. Microglia proliferation and differentiation and IRP1 expression were evaluated by immunohistochemistry.

**Results:**

Autophagy was observed in rats after SCI, accompanied by reduced latency of thermal pain, increased iron content and IRP1 and NF-κB levels in the hindlimb sensory area, hippocampus, and thalamus, and decreased Fn levels in the hindlimb sensory area. *TfR1* mRNA expression was upregulated in activated microglia. Treatment with an iron-chelating agent, or inhibitors of nitric oxide synthase or microglia suppressed microglia proliferation.

**Conclusions:**

SCI may induce intracranial iron overload, which activates microglia via NF-κB signaling. Microglia secrete inflammatory factors that induce neuronal damage and lead to CP. Treatment with an iron-chelating agent or NF-κB or microglia inhibitors can relieve CP resulting from SCI.

## Background

Around 60–90% of patients with spinal cord injury (SCI) develop central pain (CP) [1, 2], which is defined as pain hypersensitivity resulting from central nervous system injury. Pain in the lower limbs is often persistent and difficult to endure [3, 4], making CP a major problem for patients and physicians. It is thought that structural and functional remodeling of the brain after SCI can cause CP; however, the mechanisms underlying the development of CP are poorly understood [5–9]^.^

In our previous work, we used magnetic susceptibility weighted imaging to investigate the functional remodeling of the brain in SCI patients. We found that iron-overloaded regions—including the sensory regions of the cortex, thalamus, and cingutate (unpublished data)—were remodeled, implying that intracranial iron overload is involved in the pathogenesis of CP.

Intracranial iron overload plays an important role in the occurrence and development of Alzheimer’s and Parkinson’s disease and cerebral hemorrhage [10–12]. The toxicity of iron may be linked to oxidative stress injury induced by the Fenton reaction. Iron overload can activate brain microglia, which initiate and amplify neuronal damage [13]. Activated microglia also regulate the levels of cytokines such as interleukin-1β, tumor necrosis factor (TNF)-α, and nitrous oxide; the ensuing formation of reactive oxygen species and amplification of the proinflammatory cytokine cascade can lead to neuronal damage or loss [14, 15]. In addition, activated microglia may induce nuclear factor (NF)-κB signaling in neurons [16, 17].

The present study investigated the causes of intracranial iron overload and its relationship with CP pathogenesis. Our findings provide a basis for the treatment of CP following SCI through the use of iron-chelating agents and NF-κB and microglia inhibitors.

## Methods

### Experimental design

In this experiment,we selected l-arginine as the nitric oxide synthase (NOS) inhibitors, deferoxamine (DFX) as the iron chelator, and minocycline as the microglia activation inhibitors.

Female Sprague-Dawley rats (*n* = 75; 230.0 ± 15.4 g) were randomly divided into five groups (*n* = 15 each): sham operation (laminectomy only without SCI or drug treatment); control (CP rats after SCI without drug treatment); l-arginine treatment (1.5 mg/kg by intraperitoneal (i.p.) injection on the day after surgery, followed by once-weekly injections until the end of the experiment); DFX treatment (100 mg/kg by i.p. injection 1 day after surgery, followed by once-weekly injections until the end of the experiment); and minocycline treatment (45 mg/kg by i.p. injection immediately after surgery repeated over 12 h, followed by 22.5 mg/kg by i.p. injection twice daily for two consecutive days).

### Rat model of CP after SCI

Rats were anesthetized with 10% chloral hydrate (3 ml/kg) and subjected to T10 laminectomy under sterile surgical conditions by making a 3-cm incision around the T10 vertebra. The skin and muscle were cut, and the T9–T11 spine and lamina and spinal cord dorsal epidura were exposed over an area of about 2–3 mm. Using the Allen hitting method and guided by a plastic catheter, the blunt end of a 30-g stainless steel rod was dropped vertically from a height of 15 cm onto the gasket in order to induce SCI, which was confirmed by observing the twitching of the rat’s hindlimbs and tail. The incision was then sutured shut. Sham-operated animals were subjected to the same procedure but without the weight drop. After the operation, rats were placed in individual cages and administered 0.6 g lincomycin by intramuscular injection for 3 days to prevent incision infection and assisted with urination if necessary.

### Assessment of pain behavior

Following surgery, rats were observed for spontaneous pain behaviors, including trimming, scratching, licking of the hindlimbs and tail, and vocalizations.

### Determination of thermal pain hypersensitivity

The latency of thermal pain hypersensitivity was measured for each group at 2, 4, 8, 12, 16, and 24 h and then once daily after surgery. Each rat’s foot was placed on a hot plate (55 °C), and the time until the rat lifted its foot was recorded. Both feet were tested with a 3-min interval between each measurement. The procedure was repeated three times, and the average value was determined.

### Determination of cerebral cortex iron content

The iron content of the whole rat brain and various brain regions was determined by atomic absorption spectrophotometry. The whole brains or hindlimb sensory areas, thalamus, and hippocampus were collected and weighed, immersed in 20 mmol/1 HEPES buffer (1:20, *w*/*v*), and homogenized. A 30-μl volume of homogenate was mixed with an equal volume of ultrapure nitric acid and digested in a 50 °C water bath for 48 h, then diluted with 3.12 mmol/l nitrate at a 1:10 ratio. A standard curve was prepared using iron solution (50 mg/l) diluted with 5% nitric acid. The blank and actual samples were read three times at 248.3 nm.

### Determination of transferrin receptor (TfR)1 mRNA levels by real-time (RT-)PCR

*TfR1* mRNA expression in various brain regions, including the hindlimb sensory cortex, hippocampus, and thalamus (*n* = 3 rats/group) was determined by RT-PCR. RNA was extracted from tissue with TRIzol reagent (Invitrogen, Carlsbad, CA, USA) according to the manufacturer’s instructions, and 20 μg were treated with 10 U DNaseI (Takara Bio, Otsu, Japan) for 30 min at 37 °C. cDNA was synthesized using oligo dT primer, and 1 μl was added to the reaction containing 27.5 μl Real-Time PCR Master Mix(TOYOBO), 15 pmol of primers, and 7.5 pmol TaqMan probe, for a total volume of 30 μl. Primers and probes for *TfR1* and *ferritin* (*Fn*) were designed with Primer Premier 5.0 software and synthesized by Shenggong Biotechnology (Shanghai, China). The sequences were as follows: Tfrc-F, CGT GGA GAC TAC TTC CGT GC, and Tfrc-R, GCC AGA GCC CCA GAA GAT GTG; GAPDH-F, CGGCAAGTTCAACGGCACAG, and GAPDH-R CCATGGTGGTGAAGACGCCA.

### Determination of TfR1 and Fn levels in the brain by enzyme-linked immunosorbent assay (ELISA)

Tissue from the hindlimb sensory cortex, hippocampus, and thalamus of five rats was homogenized in radioimmunoprecipitation assay buffer containing protease and phosphatase inhibitors and phenylmethylsulfonyl fluoride at 4 °C. The homogenate was centrifuged at 10,000 rpm for 30 min, and the supernatant was collected and stored at −70 °C. The protein content of each sample was determined with the bicinchoninic acid assay. TfR1 and Fn levels were determined by ELISA(abcam). Each sample was prepared in triplicate, and optical density values were calculated as mean ± standard deviation.

### Determination of iron-regulatory protein (IRP)1, Fn, lactoferrin (Lf), and NF-κB levels by western blotting

IRP1, Fn, Lf, and NF-κB protein levels in the hindlimb sensory cortex of rats in each group were determined by western blotting. Briefly, 50 μg protein from brain tissue lysates were resolved by 10% non-denaturing sodium dodecyl sulfate polyacrylamide gel electrophoresis and transferred to a nitrocellulose membrane, which was confirmed by Ponceau S staining. The membrane was blocked with skim milk powder at room temperature for 2 h, followed by overnight incubation at 4 °C with rat anti-human IRP1 (Santa Cruz Biotechnology,USA) (1:100), rabbit anti-human Fn (1:100, PLLABS), rabbit anti-rat NF-κB (1:400, abcam), rabbit anti-rat LF (Santa Cruz Biotechnology,USA) (1:200), and anti-β-actin (1:100) antibodies. After three washes with tris-buffered saline containing 0.05% Tween 20, the membrane was incubated with horseradish peroxidase-conjugated secondary antibody (1:500) at room temperature. The enhanced chemiluminescence detection kit (Pierce, Rockford, IL, USA) was used to detect protein bands, which were analyzed using ImageJ software (National Institutes of Health, Bethesda, MD, USA).

### Effect of abnormal iron deposition on microglia activation

At predetermined time points, rats underwent transcardial perfusion with 4% paraformaldehyde. The brain was dissected and fixed in paraformaldehyde at 4 °C for 4–6 h, followed by immersion in 20 and 30% sucrose solutions. After the brains were saturated, they were embedded in optimal cutting temperature medium and sectioned at a thickness of 7 μm. Sections were incubated overnight with the following primary antibodies: rabbit anti-rat Anti Iba1 (WAKO,Japan) (1:200) and rat anti-human IRP1 (Santa Cruz Biotechnology,USA) (1:100). After three washes with phosphate-buffered saline, sections were incubated with biotin-conjugated secondary antibody for 90 min, washed three times, and incubated for 90 min with horseradish peroxidase-conjugated secondary antibody. After rinsing, sections were treated with diaminobenzidine (Stable DAB, Research Genetics, USA), washed with water, dehydrated, mounted, and visualized by microscopy.

### Statistical analysis

Data were analyzed with SPSS v.17.0 software. Data are presented as mean ± standard deviation. Univariate analyses were used to compare group means. *P* < 0.05 was considered as statistically significant.

## Results

### Detection of CP in a rat model

Spontaneous pain in animal models is characterized by scratching and biting below the level of injury. After SCI, rats were observed to scratch, bite, and excessively groom body parts below the level of injury, i.e., hindlimbs and tail, which was not exhibited by rats in the sham operation group.

**Fig. 1:**
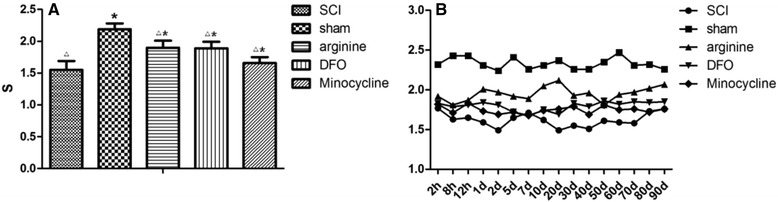
**a** Thermal pain latency in rats. *P < 0.05 vs. SCI group, △*P* < 0.05 vs. sham operation group. **b** Thermal pain latency in rats over a 90-day period

Average thermal pain latency values were as follows: 1.55 ± 0.14 s for SCI, 2.19 ± 0.09 s for sham operation, and 1.9 ± 0.11, 1.89 ± 0.10, and 1.66 ± 0.09 s for arginine, DFX, and minocycline treatment groups, respectively, (Fig. 1a). Thermal pain threshold was higher for sham-operated animals than that for the other groups (*P* < 0.05), while that of the SCI group was decreased relative to the other groups (*P* < 0.05). The sham operation group had the highest latency values, while the SCI group had the lowest values, at the time points examined (Fig. 1b).

### Determination of intracranial iron content

**Fig. 2:**
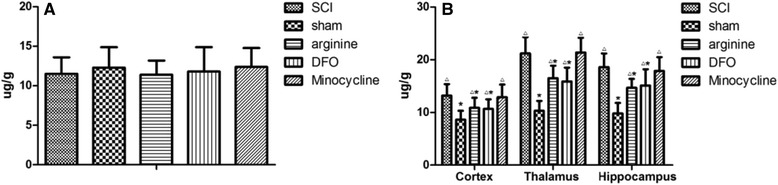
Brain iron content in rats. **a** Comparison of total brain iron content between groups. **b** Iron content in rat hippocampus, hindlimb sensory cortex, and thalamus. *P < 0.05 vs. SCI group, △*P* < 0.05 vs. sham operation group

Whole brain iron content was 11.5 ± 2.1 μg/g for SCI, 12.3 ± 2.6 μg/g for sham operation, and 11.4 ± 1.8, 11.8 ± 3.1, and 12.4 ± 2.4 μg/g for arginine, DFX, and minocycline treatment groups, respectively, (Fig. 2a). There were no differences in the whole-brain iron content among groups. However, the iron contents of the hindlimb sensory area, hippocampus, and thalamus were lower in the sham operation as compared to the other groups (*P* < 0.05), while the brain iron contents of the SCI and minocycline treatment groups were higher than those of the other groups (*P* < 0.05) (Fig. 2b and Table 1).

**Table 1:** Iron content in the sensory cortex, hippocampus, and thalamus of different groups $\left(\overline{x}\pm s\right)$

Group	SCI	sham	arginine	DFO	Minocycline
Hindlimb cortex	13.2 ± 2.2	8.6 ± 1.7	10.9 ± 1.9	10.7 ± 1.8	12.9 ± 2.4
Thalamus	21.2 ± 3.1	10.3 ± 1.9	16.5 ± 2.4	15.9 ± 2.6	21.4 ± 2.8
Hippocampus	18.6 ± 2.6	9.8 ± 2.1	14.7 ± 1.7	15.1 ± 3.1	17.9 ± 2.6

### Expression of IRP1 and NF-κB in the brain

**Fig. 3:**
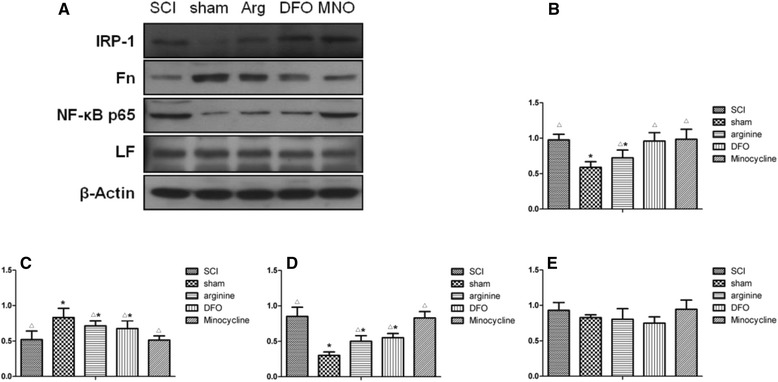
**a** Western blot analysis of IRP1, Fn, NF-κB, and LF expression in the hindlimb sensory cortex of rats. **b** The gray ratio of IRP1 and the loading control β-actin were plotted. Data represent the average of three experiments. **c**–**e** Expression levels of Fn (**c**), NF-κB (**d**), and LF (**e**). *P < 0.05 vs. SCI group; Δ*P* < 0.05 vs. sham operation group

IRP1 and NF-κB levels were lower, while Fn levels were higher, in the hindlimb sensory cortex of sham-operated rats as compared to those of other groups (*P* < 0.05) (Fig. 3). IRP1 levels were lower, while those of NF-κB and Fn were higher, in the SCI than in the sham operation, or arginine or DFX treatment groups (*P* < 0.05). These results suggest that arginine treatment decreases IRP1 and NF-κB and increases Fn levels after SCI, whereas DFX treatment decreases NF-κB and increases Fn levels but does not affect IRP1 expression. Minocycline treatment had no effect on the expression of any of these proteins, and there were no differences in LF level among groups.

### TfR1 and Fn levels in the hindlimb sensory cortex, thalamus, and hippocampus

**Fig. 4:**
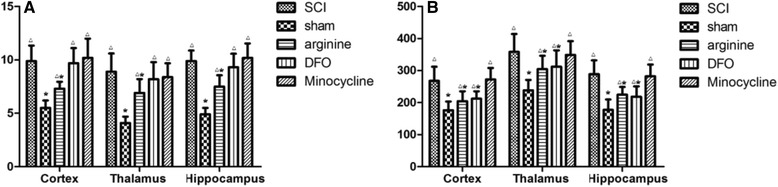
**a** TfR1 and **b** Fn expression in rat hindlimb sensory cortex, thalamus, and hippocampus, as determined by ELISA. *P < 0.05 vs. SCI group; Δ*P* < 0.05 vs. sham operation group

TfR1 levels were lower, whereas Fn levels were higher, in sham-operated animals than in the other groups (*P* < 0.05) (Fig. 4). In the SCI group, TfR1 expression was higher, whereas Fn expression was lower, than in the sham operation and arginine and DFX treatment groups (*P* < 0.05). Arginine treatment lowered TfR1 expression (*P* < 0.05) and increased Fn levels (*P* < 0.05) after SCI. DFX treatment increased Fn levels (*P* < 0.05) but had no effect on TfR1 expression, while minocycline had no effect on TfR1 or Fn levels (*P* > 0.05).

### TfR1 gene expression in the hindlimb sensory cortex, thalamus, and hippocampus

**Fig. 5:**
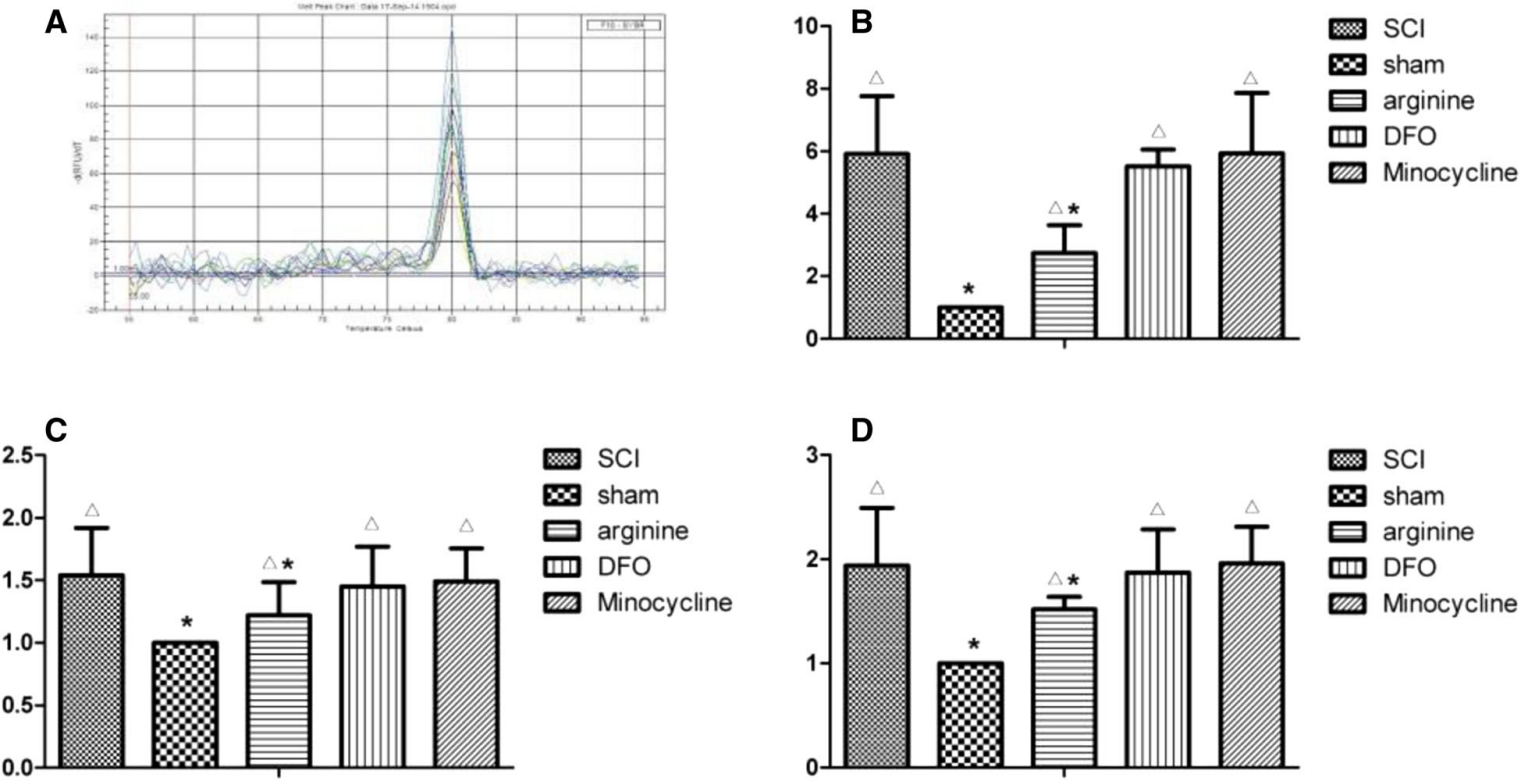
**a**–**d** *TfR1* gene expression in rat hindlimb sensory cortex, thalamus, and hippocampus. Melting curve of *TfR1* (**a**) and expression levels in rat hindlimb sensory cortex (**b**), hippocampus (**c**), and thalamus (**d**). *P < 0.05 vs. SCI group; Δ*P* < 0.05 vs. sham operation group

*TfR1* gene expression in the rat hindlimb sensory area, thalamus, and hippocampus was determined by RT-PCR (Fig. 5). *TfR1* levels in the various brain regions were lower in sham-operated rats as compared to those in the other groups (*P* < 0.05), but were higher in the SCI than in the sham and arginine treatment groups (*P* < 0.05). These results indicate that arginine and DFX treatment decrease *TfR1* gene expression after SCI, with the former showing a greater effect.

### Microglia distribution and IRP1 expression in the brain following SCI

**Fig. 6:**
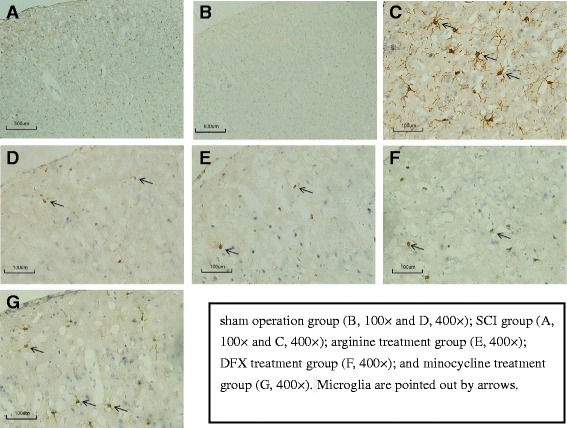
**a**–**g** Immunohistochemical analysis of microglia distribution in rat hindlimb sensory cortex

**Fig. 7:**
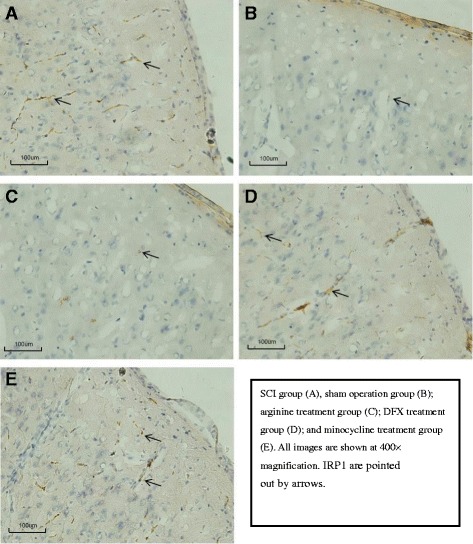
**a**–**e** Immunohistochemical analysis of IRP1 expression in rat hindlimb sensory cortex

The number (Fig. 6a, b) and activation (Fig. 6c, d) of microglia in the hindlimb sensory cortex was markedly increased after SCI. Treatment with arginine, DFX, and minocycline decreased microglia number and activation (Fig. 6e–g). Accordingly, the number of IRP1-positive cells was increased after SCI (Fig. 7a, b), an effect that was abrogated in the presence of arginine (Fig. 7c).

## Discussion

CP is also known as central dysesthesia syndrome [18] and is a common complication after SCI, hindering patients’ rehabilitation and diminishing their quality of life [19]. One study reported that 40% of SCI patients were willing to sacrifice sexual, gut, and bladder functions for a reduction in pain [20]. Since the etiology of CP after SCI is not well understood, there are no means of effectively diagnosing or treating this condition. Currently available treatments include physical therapy, drugs (non-steroidal anti-inflammatory analgesics, anticonvulsants, antidepressants, or opioids), and surgery [21]. These methods are only effective in 20–30% of cases [22]. Moreover, drugs are frequently associated with side effects and can lead to addiction. Blocking nerve fibers at the posterior root of the spinal nerve by nerve root cutting or anhydrous alcohol injection may have analgesic effects; however, this method is imprecise and is associated with a high rate of postoperative pain recurrence as well as organ dysfunction.

It has been suggested that CP following SCI arises from imbalances in the sensory pathway [23] or inhibitory and excitatory receptors [24, 25]; in addition, pattern theory [26] and the central nervous system immune response have also been proposed as explanations [27]. Some brain regions including the somatic sensory center, thalamus, and limbic system undergo structural and functional remodeling after SCI to compensate for the loss of sensory function; this may be an underlying cause of CP [5–9]. Patients with partial spinal cord transection may also experience pain in the distal part of the injured spine; although, pain severity is not directly related to whether or not there is total injury [28, 29], suggesting that the source of pain is likely proximal to the injury site. In this study we used Allen’s weight-drop method [30] to establish an SCI model. Rats with CP were treated with DFX, arginine, or minocycline, which reduced the excessive grooming behavior indicative of CP in SCI rats while increasing the thermal pain threshold.

Intracranial iron is involved in RNA and protein synthesis in the brain as well as myelination and dopamine production. However, excessive iron in the brain has been linked to neurodegenerative disorders [31, 32] due to its role in the generation of hydroxyl free radicals via the Fenton reaction [33] (Fe^2+^ + H_2_O_2_ → Fe^3+^ · OH + OH^−^), which causes oxidative stress-induced damage to cells and tissue. The redistribution of intracranial iron and iron deposition in specific brain regions has been observed in some neurodegenerative diseases such as Alzheimer’s, Parkinson’s, and Huntington’s disease and Hallervorde-Spatz syndrome [34–36]. Brain iron levels are also elevated by physical stress, including heat and exercise stress and seasickness [37, 38]. Recent studies have found mutations in genes associated with brain iron metabolism, strongly suggesting that increased brain iron levels is a causative factor in some neurodegenerative diseases [39].

We used atomic absorption spectrophotometry to determine brain iron content and found that iron levels in the thalamus, hippocampus, and hindlimb sensory area were elevated for up to 12 weeks in all SCI groups relative to sham-operated animals, similar to what is observed in some neurodegenerative disorders. These results suggest an association between retention of iron in specific brain structures and CP.

Iron metabolism in the brain depends on the expression of various iron metabolism proteins. The passage of iron through the blood-brain barrier and its uptake by neurons is mainly mediated by the Tf/TfR interaction [40–44]. We found that *TfR1* expression in the hindlimb sensory cortex, hippocampus, and thalamus was increased in all SCI rats, indicating that an increase in iron uptake via the Tf/TfR pathway may underlie iron overloading. Fn is a natural iron chelator that is widely expressed in neurons and glia in humans and rodents. It is the major form of brain iron storage, accounting for one-third to three-fourth of all iron stored in the brain [45]. Fn can be of the H or L type [46, 47]; the former is involved in the rapid uptake and reuse of iron, while the latter is associated with long-term iron storage [48]. Although iron levels are increased in Parkinson’s and Alzheimer’s disease, there is no corresponding increase in Fn levels [49], which is known to limit iron-induced brain damage [42]. Here, we found that Fn expression was decreased in the thalamus, hippocampus, and hindlimb sensory area of rats in the SCI as compared to the control group, indicating that iron storage capacity in these brain regions was impaired in the CP model, which may have resulted in an increase in free iron content. Fn and TfR expression is mainly regulated by the iron response element/IRP system. We found that IRP1 levels in the thalamus, hippocampus, and hindlimb sensory area of rats were elevated by SCI, corresponding to increased TfR and decreased Fn l expression. However, LF levels in these brain regions were unaffected by SCI, suggesting that LF-mediated iron uptake is not involved in intracranial iron overloading and CP following SCI.

DFX is an iron chelator that can pass through the blood-brain barrier and accumulate in the brain parenchyma, preventing the release of iron from Fn and thereby reducing oxidative damage caused by iron overload [26]. In this study, DFX treatment abrogated the increase in iron levels in the hippocampus, hindlimb sensory area, and thalamus of rats resulting from SCI.

NF-κB has been shown to be activated by Fe^2+^ in macrophages [50, 51], and in turn activates microglia [50]; application of an NF-κB inhibitor can abolish this effect and limit the damage to neurons caused by SCI [52, 53]. We found that NF-κB levels were elevated in the hindlimb sensory area of rats after SCI; however, this effect was mitigated by treatment with DFX or NF-κB inhibitor.

The presence of activated microglia is a hallmark of central nervous system diseases characterized by the loss of neurons, such as Parkinson’s and Alzheimer’s disease [54]. Long-term use of anti-inflammatory drugs that target the cytokines released by microglia can reduce the rate of AD and PD by about 50% [55, 56]. In animal models of neuropathic pain, it was found that peripheral nerve injury activates microglia; conversely, inhibiting microglia activation reduced the occurrence of hyperalgesia and evoked pain. Microglia can capture free iron in the brain and store these ions in Fn molecules. Injection of FeCl_2_ into the hippocampus of rats delayed injury to neurons, but also induced the activation of microglia [57]. In the present study, minocycline treatment suppressed the activation of microglia in the hindlimb sensory area of rats after SCI.

Analysis of behavioristics and the intracranial iron content, however, revealed that the NOS inhibitors treatment was slightly better compared with iron chelator and the microglia activation inhibitors, although this difference was not statistically significant. One could make a feasible explanation that remodeling of the brain after SCI contains multiple mechanisms and factors, and several pathways could be inhibited at upstream by blocking NOS pathway.

## Conclusions

The results presented here indicate that after SCI, activation of IRP can lead to intracranial iron overload, which activates microglia via the NF-κB signaling pathway. The proinflammatory cytokines secreted by these microglia causes neuronal damage and loss, leading to CP. This effect can be abrogated by treatment with an iron-chelating agent, NF-κB inhibitor, or microglia inhibitor, suggesting that these agents can effectively relieve CP in SCI patients. There are mechanisms of CP out of scope of this article; therefore, further research is required.

## Abbreviations

CP: Central pain
DFX: Deferoxamine
Fn: Ferritin
IRP1: Iron-regulatory protein1
Lf: Lactoferrin
NOS: Nitric oxide synthase
SCI: Spinal cord injury
TfR1: Transferrin receptor 1
